# Nuclear organization of nucleotide excision repair is mediated by RING1B dependent H2A-ubiquitylation

**DOI:** 10.18632/oncotarget.16142

**Published:** 2017-03-11

**Authors:** Shalaka Chitale, Holger Richly

**Affiliations:** ^1^ Laboratory of Molecular Epigenetics, Institute of Molecular Biology, Mainz, Germany, Ackermannweg, Mainz, Germany; ^2^ Faculty of Biology, Johannes Gutenberg University, Mainz, Germany

**Keywords:** nucleotide excision repair, DNA repair, ubiquitin, chromatin, nuclear dynamics

## Abstract

One of the major cellular DNA repair pathways is nucleotide excision repair (NER). It is the primary pathway for repair of various DNA lesions caused by exposure to ultraviolet (UV) light, such as cyclobutane pyrimidine dimers (CPDs) and 6-4 photoproducts. Although lesion-containing DNA associates with the nuclear matrix after UV irradiation it is still not understood how nuclear organization affects NER. Analyzing unscheduled DNA synthesis (UDS) indicates that NER preferentially occurs in specific nuclear areas, viz the nucleolus. Upon inducing localized damage, we observe migration of damaged DNA towards the nucleolus. Employing a LacR-based tethering system we demonstrate that H2A-ubiquitylation via the UV-RING1B complex localizes chromatin close to the nucleolus. We further show that the H2A-ubiquitin binding protein ZRF1 resides in the nucleolus, and that it anchors ubiquitylated chromatin along with XPC. Our data thus provide insight into the sub-nuclear organization of NER and reveal a novel role for histone H2A-ubiquitylation.

## INTRODUCTION

Nucleotide excision repair (NER) is one of the major DNA repair pathways and handles various lesions such as cyclobutane pyrimidine dimers (CPDs) and 6-4 photoproducts, which occur after exposure to ultraviolet (UV) light [1]. Defects in NER cause genetic disorders such as *Xeroderma pigmentosum*, which constitutes hypersensitivity to sunlight and a predisposition for skin cancer [2]. Mammalian NER recognizes DNA lesions by two different pathways. Transcription-coupled NER (TC-NER) is limited to regions of active transcription, where RNA Polymerase II stalling elicits the DNA damage response. In contrast, transcription-independent recognition of lesions is handled by global genome NER (GG-NER) [3, 4]. During GG-NER, lesions are detected by the damage recognition factors XPC and DDB2. XPC specifically recognizes structures that distort the DNA double-helix, binds damaged DNA, and rapidly dissociates upon triggering NER [5–7]. Efficient recognition of CPDs and 6-4 photoproducts also requires DDB2 (XPE) [8–12]. DDB2 along with DDB1, the RING-domain proteins RBX1 or RING1B, and either of the scaffold proteins CUL4A or CUL4B forms E3 ubiquitin ligase complexes (UV-DDB-CUL4A/B and UV-RING1B). These complexes catalyze the mono-ubiquitylation of histones H2A, H3 and H4 as well as the polyubiquitylation of XPC [13–16].

Studies of DNA repair have shown that nuclear positioning and migration of the damaged DNA to specific repair centers is a central component of many repair pathways [17–20]. In mammalian cells, nuclear organization during double-strand break (DSB) repair affects chromosome translocations [21] and pathway choice [22]. During NER, the damage recognition factor DDB2 promotes local chromatin decondensation [23] and NER seems to involve large-scale chromatin rearrangements [24]. Additionally, it has been recently shown that heterochromatin impedes CPD removal, and this process is enabled by DDB2 [25]. Although lesion-containing DNA associates with the nuclear matrix after UV irradiation [26] it is less well understood how nuclear organization affects NER.

Another important feature of DNA repair is H2A-ubiquitylation. At DSBs, ubiquitylation of H2A is carried out by the E3 ligases RNF168, RNF8, and RING1B, which facilitate signaling and accumulation of repair proteins [27–30]. Furthermore, it was demonstrated that the RING1B-catalyzed ubiquitylation through Polycomb-repressive complex 1 (PRC1) mediates DSB-induced gene silencing, highlighting an additional function of H2A-ubiquitylation in DNA repair [30]. During NER H2A-ubiquitylation is catalyzed by the E3 ligase RNF8, the UV-DDB-CUL4 and UV-RING1B complexes [14, 31–34]. We have recently shown that ZRF1 is an essential factor in NER. ZRF1 binds the H2A-ubiquitin mark catalyzed by the UV-RING1B complex, and its presence at damaged chromatin depends on the recognition factor XPC [34].

Here we report that H2A-ubiquitylation via the UV-RING1B complex repositions chromatin close to the nucleolus. We provide further evidence that ZRF1 resides in the nucleolus and that H2A-ubiquitylation and its recognition by ZRF1 facilitate nucleolar DNA repair.

## RESULTS

### NER is partially routed to the nucleolus and involves reorganization of chromatin

**Figure 1:**
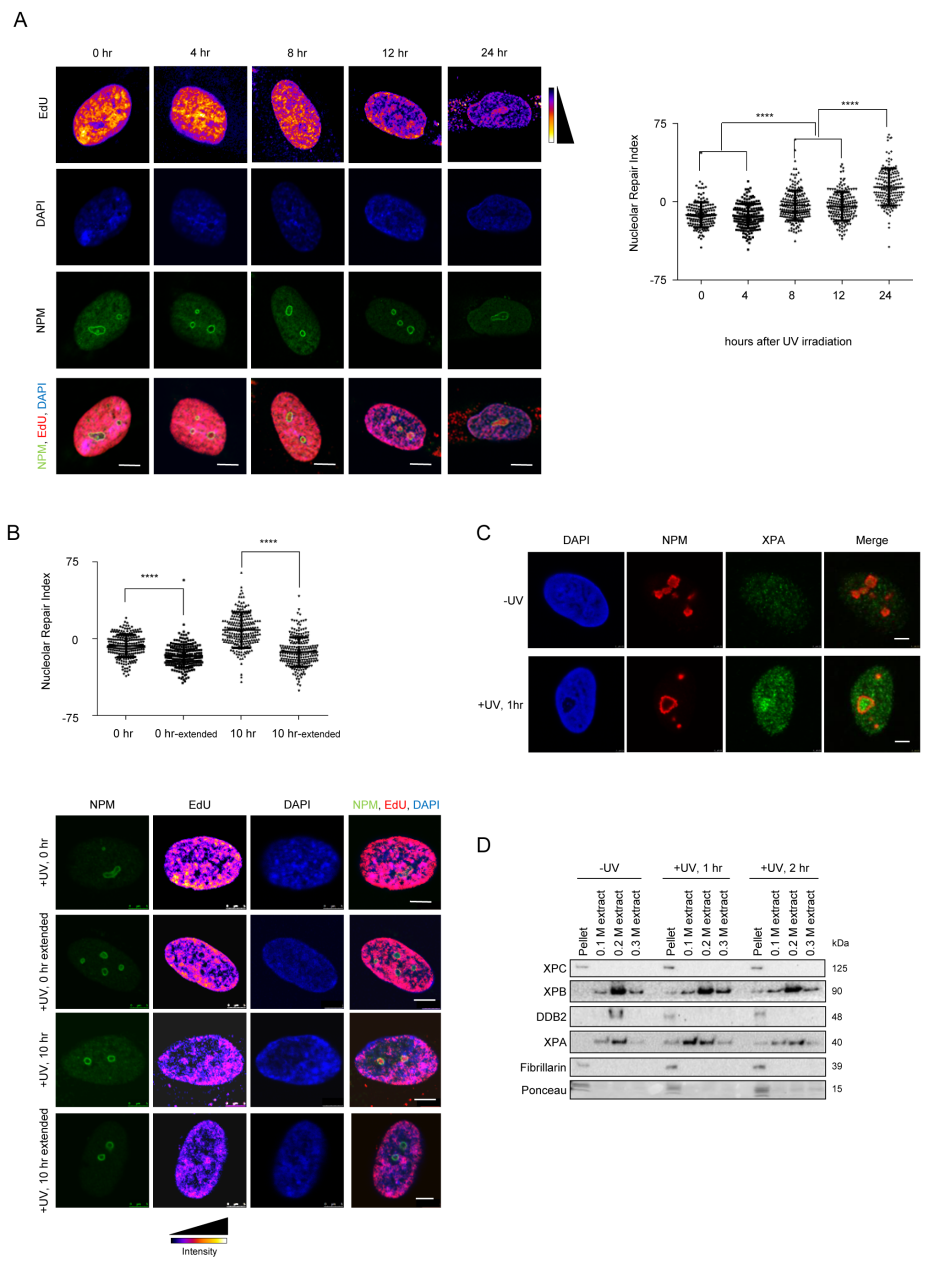
The nucleolus is a site of accumulation of NER proteins as well as active DNA repair **A**. NER occurs in repair foci that colocalize with nuclophosmin (NPM-a nucleolar protein). (Left panel) Representative images of active repair foci indicated by EdU incorporation, at the indicated time points post UV exposure. Cells are co-stained with NPM to show nucleolar overlap of repair foci. Scale bar: 5μm. (Right panel) The graph shows the Nucleolar Repair Index (NRI) values of nucleoli from ≈100 non-S phase cells at the indicated time points post UV exposure. Statistical significance was calculated using a Mann-Whitney test. **B**. DNA repaired in the nucleolus is later redistributed throughout the nucleus. (Top panel) The graph shows the Nucleolar Repair Index (NRI) values of nucleoli from ≈100 non-S phase cells at the indicated time points post UV exposure, with or without extended repair up to 24 hours (extended) in EdU free medium. Statistical significance was calculated using a Mann Whitney test. (Bottom panel) Representative images of active repair indicated by EdU incorporation, at the indicated time points post UV exposure, stained either immediately after incorporation, or after extended repair in EdU free medium up to 24 hrs (extended). Scale bar: 5μm. **C**. XPA accumulates in the nucleolus post UV exposure. Immunofluorescence images showing staining of NPM and XPA in pre-extracted cells unexposed to UV, or 1 hour after exposure to a 20J/m^2^ UV dose. Pre-extraction washes off unbound nuclear and cytoplasmic proteins and enables visualization of chromatin-associated proteins. In UV exposed cells, nucleolar XPA is seen in 73.57% ±10.13 cells. 60-80 cells were counted per replicate. *n* = 3. Scale bar: 5μm. **D**. Nucleolar NER proteins are present in the chromatin fraction post UV exposure. Western blot showing sequential salt extraction fractions of nucleoli at the indicated timepoints post UV exposure. The pellet consists of the chromatin bound fraction. **E**. (Left panel) Immunofluorescence images showing EdU incorporation in MRC5 cells subjected to DNA damage through a micropore of a 3 μm diameter. Cells were incubated with EdU and fixed and stained at the indicated time points after UV damage. Nuclei are shown by DAPI staining, and nucleoli are marked by Nucleophosmin (NPM). Scale bar: 5μm. (Right top panel) Quantification of absolute nuclear intensity of the EdU signal after incubation for the indicated timepoints after micropore UV damage. 70 cells measured per timepoint. (Right bottom panel) The graph shows the Nucleolar Repair Index (NRI) values of nucleoli from ≈70 non-S phase cells at the indicated time points post UV exposure. Statistical significance was calculated using a Mann-Whitney test.

In order to determine whether NER, similar to other DNA repair pathways, occurs in so called ”repair factories” we studied the nuclear distribution of repair. We visualized unscheduled DNA synthesis (UDS) in fibroblasts to determine whether DNA repair shows a bias in nuclear distribution. In order to analyze the distribution at various stages of repair, we pulsed the cells with EdU for 2 hours at various time points post UV exposure and measured the EdU incorporation in non S-phase cells (Supplementary Figure 1A). Immediately after UV exposure, DNA repair occurred uniformly throughout the nucleus with no discernible patterns. However, at later time points after UV exposure, we observed repair occurring in specific nuclear foci (Figure 1A). These foci resembled nucleoli in size and number, and thus we performed a co-staining with nucleophosmin (NPM- a marker for the nucleolus). We found that the repair foci indeed overlapped with nucleoli (Figure 1A). In order to measure the share of repair occurring in the nucleolus, we measured the mean EdU intensity in the nucleolus, nucleoplasm as well as in the whole nucleus. Using these values we calculated the Nucleolar Repair Index (NRI) as (Nucleolus^mean^- Nucleoplasm^mean^)/Nucleus^mean^ ×100, where a positive NRI reflects an enrichment of DNA repair in the nucleolus. Interestingly, 8 hours after UV irradiation we started to observe an enrichment of EdU incorporation in the nucleolus as compared to the nucleoplasm, reflected by a positive NRI, which increased with time reaching its maximum 24 hours post irradiation (Figure 1A). In non-irradiated cells we did not detect any EdU incorporation confirming that the incorporation was indeed a consequence of active DNA repair (Supplementary Figure 1A). Additionally, we analyzed the images to determine whether the observed nucleolar EdU enrichment is a consequence of the underlying chromatin structure, specifically due to the presence of perinucleolar heterochromatin. We normalized the EdU signal by the DAPI signal, giving us a normalized image for EdU per DNA amount. We then measured the mean EdU intensity in the nucleolus, nucleoplasm as well as in the whole nucleus in the normalized image. Using these values we calculated the Nucleolar Repair Index (NRI). The NRI showed a similar increase in nucleolar signal as compared to the non-normalized EdU signal (Supplementary Figure 1E), thus demonstrating that the enrichment seen is independent of the underlying chromatin structure.

Next, we wanted to determine if the measured nucleolar EdU incorporation is due solely to the repair of perinucleolar heterochromatin or whether it predominantly reflects repair of non-nucleolar chromatin. To this end, we EdU-pulsed cells for 2 hours, at 0 and 10 hours after UV irradiation, and subsequently allowed the repair to proceed in EdU-free medium up to 24 hours. We chose the 10 hour time point, as we had observed an overall sufficient repair signal and enrichment of nucleolar repair at this time (Figure 1A). We noticed that, 12 hours after changing to EdU-free medium, the nucleolar enrichment of the EdU signal was lost and that it was once again evenly distributed within the nucleus for both the 0 and 10 hour time points (Figure 1B). This re-distribution of the repaired DNA suggests a dynamic relationship between the site of repair and the subsequent nuclear positioning of the repaired DNA. Notably, the re-distribution of repaired DNA occurred even for DNA repaired immediately after UV damage, reflected by a decrease in the NRI. This further implies that nucleolar repair occurs throughout the repair process, but that at later time points the majority of repair occurs in the nucleolus.

To further confirm whether the nucleolus acts as a repair center, we investigated whether the endogenous NER proteins relocated to the nucleolus upon UV irradiation. We observed a distinct enrichment of XPA, a critical NER protein [1] in the nucleolus of pre-extracted U2OS and MRC5 cells, starting at 1 hour after UV irradiation (Figure 1C and Supplementary Figure 1D). Furthermore, we assessed the presence of selected DNA repair factors in purified nucleoli before and after UV irradiation (Figure 1D, Supplementary Figure 1B, 1C and 1F). We found repair proteins such as XPC and XPB in nucleoli even in unexposed cells, while XPA levels increased post UV exposure (Supplementary Figure 1F). In addition, we tested whether the proteins found in the nucleolus were also chromatin bound. To this end, we extracted purified nucleoli with increasing salt concentrations (Figure 1D). We found XPC, XPB, DDB2 and XPA associated to chromatin in nucleoli of irradiated cells.

Finally, we wanted to assess if damaged DNA also translocates to the nucleolus. To this end, we inflicted localized damage on the cells by irradiation through a micropore membrane. We incubated these locally damaged cells in EdU for various time points post UV. We then calculated the total EdU intensity as well as the Nucleolar Repair Index (NRI) for these cells. As expected, we observed that the total amount of EdU incorporated increased with increasing times of incubation. Thus, we were able to observe repair occurring until 24 hours post UV exposure (Figure 1E). However, when we calculated the NRI for the same cells, we observed a decrease of the NRI over time. At early time points, DNA repair occurred in the nucleolus and therefore a maximum EdU signal was observed within the nucleolus. However, at later time points, the majority of the DNA was already repaired, and thus no longer present in the nucleolus. This is reflected by the observed decrease in the NRI (Figure 1E). Taken together our data suggest that GG-NER is carried out in part at the nucleolus and that it involves re-localization of the damaged DNA.

### DDB2 causes repositioning of chromatin to the vicinity of the nucleolus

**Figure 2:**
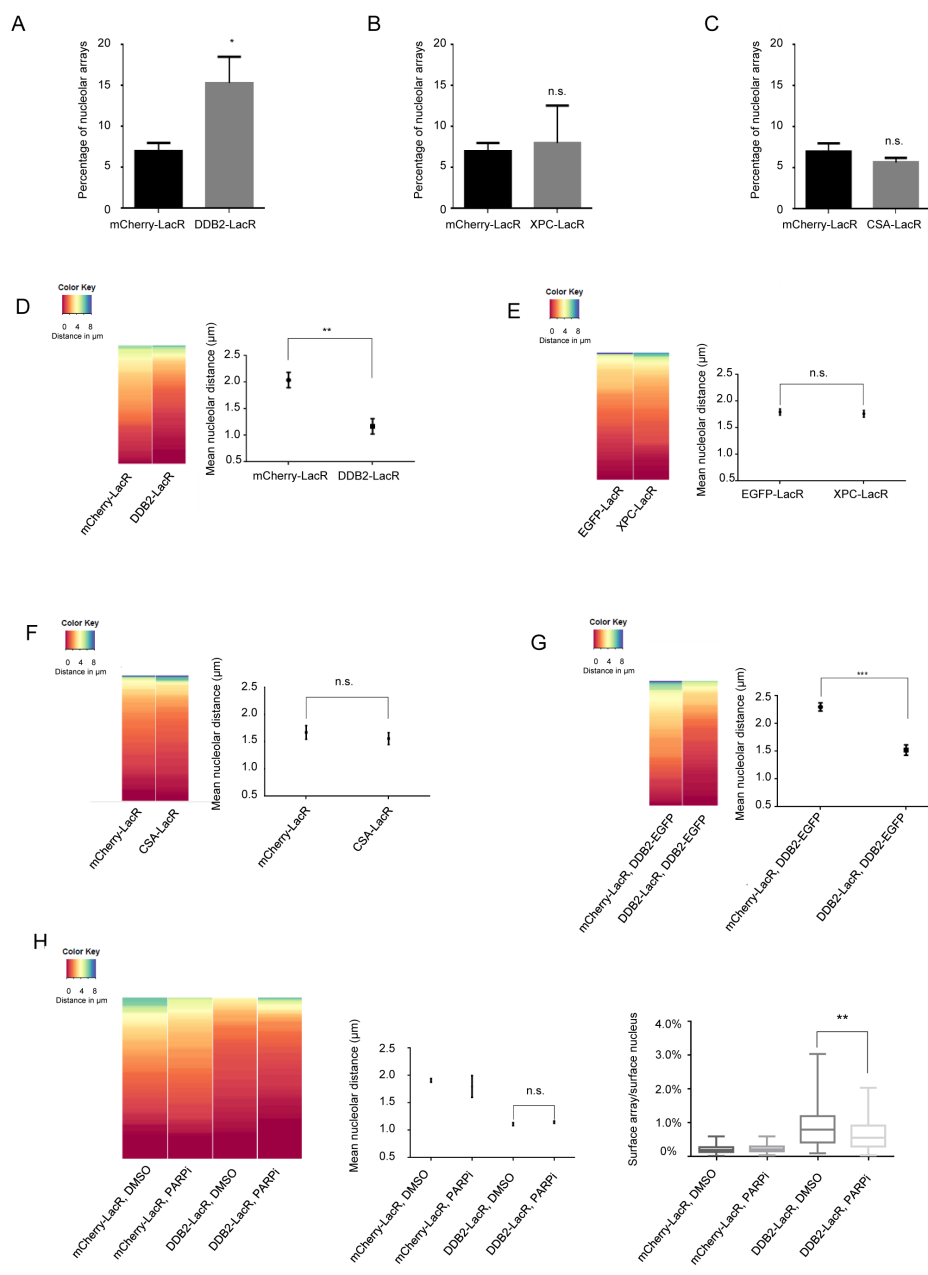
DDB2 tethering causes repositioning of a LacO array to the nucleolus **A**. Quantification of nucleolar arrays (distance from nucleolus = 0μm) in control (mCherry-LacR) and DDB2 (DDB2-LacR) tethered arrays. 90-100 cells were counted per experiment. The graph shows the mean ±SD. (*n* = 3). Significance was determined by an unpaired t-test. **B**. Quantification of nucleolar arrays (distance from nucleolus = 0μm) in control (mCherry-LacR) and XPC (XPC-LacR) tethered arrays. 90-100 cells were counted per experiment. The graph shows the mean ±SD. (*n* = 3). Significance was determined by an unpaired t-test. **C**. Quantification of nucleolar arrays (distance from nucleolus = 0μm) in control (mCherry-LacR) and CSA (CSA-LacR) tethered arrays. 90-100 cells were counted per experiment. The graph shows the mean ±SD. (*n* = 3). Significance was determined by an unpaired t-test. **D**. (Left panel) Heat map showing the distribution of the nucleolar distance of the array in 100 cells. Control arrays (mCherry-LacR) and DDB2 tethered arrays (DDB2-LacR) were analyzed. The distribution of the control array differs significantly from the DDB2 array as judged by a KS test. (p value≤ 0.0001). (Right panel) Mean nucleolar distance of the array in control and DDB2 conditions. The mean nucleolar distance of each replicate was calculated from measurements of the nucleolar distance in 100 cells. The graph shows the average of the means from 3 independent experiments ±SD. Statistical significance was determined using an unpaired t-test. **E**. Tethering of XPC does not cause repositioning of the LacO array. (Left panel) Heat map showing the distribution of the nucleolar distance of the array in 100 cells. Control arrays (mCherry-LacR) and XPC tethered arrays (XPC-LacR) were analyzed. The distribution of the control array is not significantly different compared to the XPC tethered array as judged by the KS test. (Right panel) Mean nucleolar distance of the array in control and XPC conditions. The mean nucleolar distance of each replicate was calculated from measurements of nucleolar distance in 100 cells. The graph shows the average of the means from 3 independent experiments ±SD. Statistical significance was determined using an unpaired t-test. **F**. Tethering of CSA does not cause repositioning of the LacO array. (Left panel) Heat map showing the distribution of the nucleolar distance of the array in 100 cells with control arrays (mCherry-LacR) and CSA tethered arrays (CSA-LacR). Distribution of the control array does not significantly differ from the CSA-tethered array as judged by KS test. (Right panel) Mean nucleolar distance of the array in control and CSA conditions. The mean nucleolar distance of each replicate was calculated from measurements of the nucleolar distance in 100 cells. The graph shows the average of the means from 3 independent experiments ±SD. Statistical significance was determined using an unpaired t-test. **G**. Repositioning of the array is specifically an effect of local DDB2 tethering. DDB2-EGFP was expressed in cells with mCherry-LacR and DDB2-LacR tethered arrays (Left panel) Heat map showing the distribution of the distance between the array and the nucleolus in 100 cells. Expression of DDB2-EGFP does not affect the distribution of the mCherry-LacR array. The distribution of the control array differs significantly from the DDB2 array as judged by a KS test. (p value≤ 0.0001). (Right panel) Mean nucleolar distance of the array in the corresponding cells. The mean nucleolar distance of each replicate was calculated from measurements of the nucleolar distance in 100 cells. The graph shows the average of the means from 3 independent experiments ±SD. Statistical significance was determined using an unpaired t-test. **H**. DDB2-LacR mediated repositioning of the array is not dependent on DDB2 mediated chromatin decondensation. DDB2-LacR cells were subjected to treatment with either DMSO (control) or PARP inhibitor (PARPi). (Left panel) Heat map showing the distribution of the distance between the array and the nucleolus in 100 cells. Treatment with PARPi does not affect the repositioning of the array. The distribution of the DDB2 array in the DMSO treated versus PARPi treated cells does not differ. (Middle panel) Mean nucleolar distance of the array in the corresponding cells. The mean nucleolar distance of each replicate was calculated from measurements of the nucleolar distance in 100 cells. The graph shows the average of the means from 3 independent experiments ±SD. Statistical significance was determined using an unpaired t-test. (Right panel) The graph shows the distribution (Min to Max) of percentage of nuclear area occupied by the specified tethered array. Array size was measured in 50-100 cells from two independent experiments. Statistical significance was assessed by an unpaired t-test.

As lesion recognition is essential for subsequent repair, we wanted to determine if lesion recognition led to translocation of DNA to the nucleolus for repair. To directly assess the effect of binding of recognition proteins to DNA, we used a lactose repressor (LacR) based system for tethering proteins to a defined chromosome region *in vivo* [35, 36]. We tethered mCherry-LacR fusions of DDB2, XPC and CSA to a heterochromatic locus in human U2OS 2-6-3 cells, containing 200 copies of a LacO containing cassette (total array size ≈ 4Mbp) [35]. We expressed mCherry-LacR-fusion and mCherry-LacR proteins in the U2OS 2-6-3 cell line and analyzed the nuclear positioning of the array. We observed a significant increase in the number of nucleolar arrays (distance from nucleolus 0 μm) upon tethering of DDB2 (Figure 2A), while tethering of XPC or CSA did not show a significant effect (Figure 2B and 2C). Representative images and the corresponding nucleolar distance are shown in Supplementary Figure 2A. Our UDS data suggested the nucleolus to be a site of active DNA repair. The LacO tethering system however, mimics the recognition and subsequent relocalization of chromatin to the nucleolus. In order quantify this dynamic process, we additionally determined if the tethered array was positioned overall closer to the nucleolus. The nucleolar distance was measured as the distance of the array to the closest nucleolus. Distances were measured for 100 randomly picked transfected cells per sample [37, 38], and the mean nucleolar distance from at least 3 independent experiments was calculated. Tethering of DDB2 caused a relocation of the DNA array and positioned it closer to the nucleolus (mean ≈1μm). In comparison, the untethered control (mean ≈2μm) showed a random distribution of the array in agreement with a previous report [39] (Figure 2D). Tethering of XPC-LacR or CSA-LacR did not significantly relocate the array to the vicinity of the nucleolus (Figure 2E and 2F). It has been previously reported that tethered DDB2 recruits GFP tagged DDB1 and CUL4A [23]. We additionally confirmed the functionality of tethered DDB2 by staining with DDB1 antibodies, as well as by showing recruitment of XPC-GFP to the array (Supplementary Figure 2B). In addition we verified that tethered CSA-LacR co-localized with its binding partner DDB1 [40, 41] (Supplementary Figure 2D). Expression of mCherry-LacR-DDB2 also results in excess of unbound nuclear protein. To confirm that the repositioning is indeed a consequence of the tethered DDB2, and not an effect of global DDB2 overexpression, we expressed DDB2-EGFP in cells containing mCherry-LacR and DDB2-LacR tethered arrays. Overexpression of DDB2-EGFP caused no relocation of the control array and did not impair repositioning by the tethered DDB2-LacR (Figure 2G). Indirect tethering of DDB2-EGFP through a GFP binding protein (GBP)-LacR system [42–44] also led to a similar re-localization (Supplementary Figure 2C). It has been previously shown that tethering of DDB2 leads to decondensation of a chromatin array in a PARP dependent manner [45]. Thus, we wanted to determine if the observed repositioning is linked to the DDB2 dependent decondensation. To this end, we incubated mCherry-LacR or DDB2-LacR cells with IPTG, followed by an IPTG washout and 16 hour incubation with either DMSO or a PARP inhibitor (KU 0058948). We then fixed the cells and measured the nucleolar distance as well as the array size. As shown before, treatment with a PARP inhibitor inhibited DDB2 mediated decondensation as observed by a decrease in the array size (Figure 2H). However, PARPi treatment did not have any effect on the repositioning of the array. In both DMSO and PARPi treated cells, the DDB2-LacR array showed a significantly reduced nucleolar distance (Figure 2H). Thus the DDB2 mediated repositioning is independent of the decondensation of the array. Altogether, these data suggest that specifically DDB2 is important for repositioning the array close to the nucleolus, and this function is not dependent on PARP activation. Importantly, the repositioning of the array occurs only through components of the GG-NER pathway, which is in agreement with the later time points at which an enrichment of nucleolar repair is observed [46, 47]

### Nucleolar repositioning requires presence of a functional UV-RING1B complex and H2A-K119 ubiquitylation

**Figure 3:**
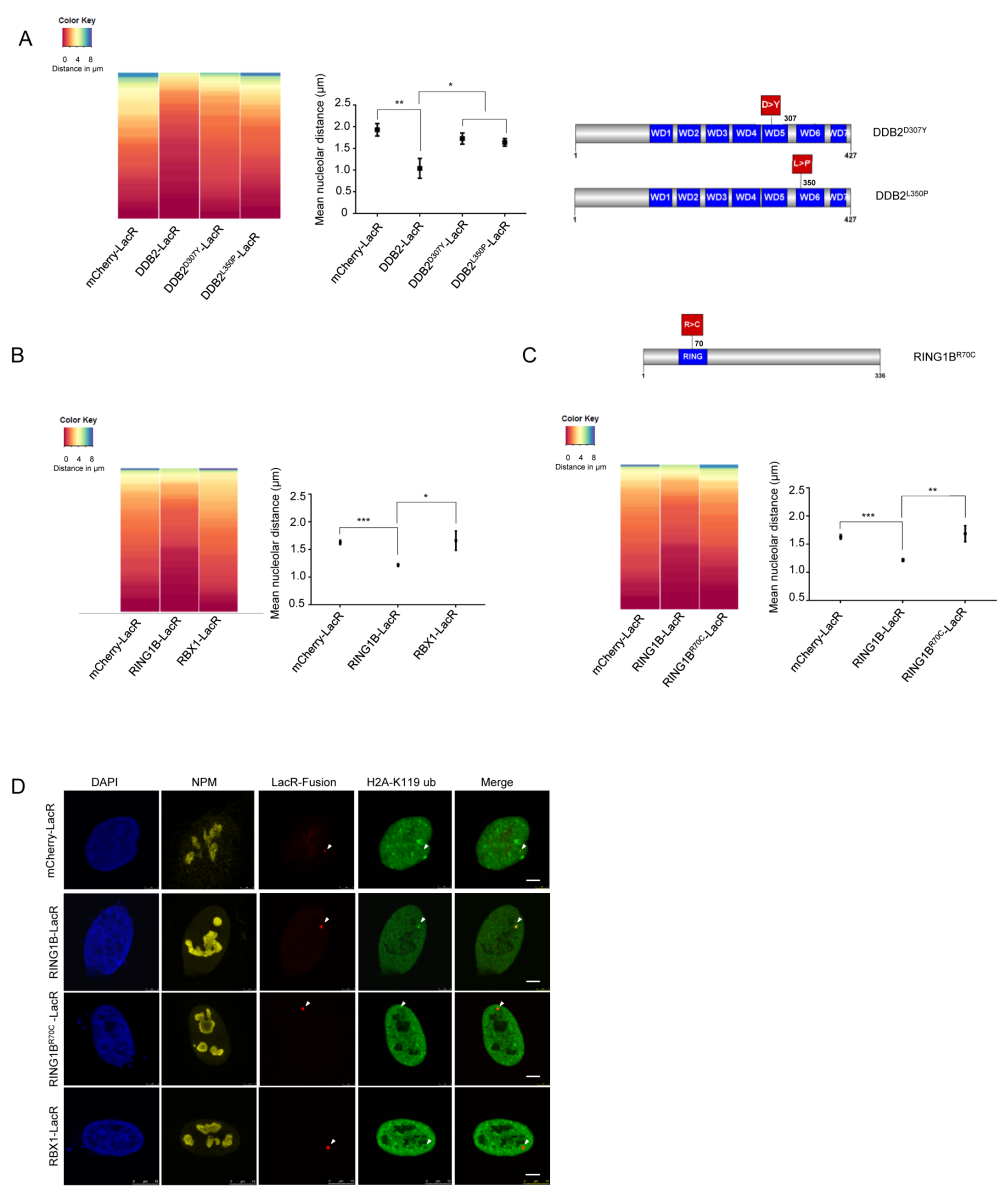
Repositioning of the array is dependent on formation of a functional UV-DDB complex **A**. Tethering of DDB2 mutants does not lead to repositioning of the LacO array. (Left panel) Heat map showing the distribution of the nucleolar distance of the array in 100 cells. Distances were measured in control arrays (mCherry-LacR) and DDB2-LacR, DDB2^D307Y^-LacR and DDB2^L350P^-LacR tethered arrays. Relocalization of DDB2 differs significantly from the control array as judged by a KS test. (p value≤ 0.0001). Tethering DDB2^D307Y^-LacR or DDB2^L350P^-LacR does not differ significantly from the control. (Middle panel) Mean nucleolar distance of the array in control, DDB2-LacR, DDB2^D307Y^-LacR and DDB2^L350P^-LacR tethered conditions. The mean nucleolar distance of each replicate was calculated from measurements of nucleolar distance in 100 cells. The graph shows the average of the means from 3 independent experiments ±SD. Statistical significance was determined using an unpaired t-test. (Right panel) Graphical representation of the DDB2 point mutations used in this study. **B**. RING1B but not RBX1 tethering leads to repositioning of the array. (Left panel) Heat map showing the distribution of nucleolar distance of the array in 100 cells. Control arrays (mCherry-LacR), RING1B-LacR and RBX1-LacR tethered arrays were analyzed. The distribution of the RING1B-LacR array differs significantly when compared to the control array as judged by the KS test (p value≤ 0.001). (Right panel) Mean nucleolar distance in mCherry-LacR, RING1B-LacR and RBX1-LacR tethered arrays. The mean nucleolar distance of each replicate was calculated from measurements of nucleolar distance in 100 cells. The graph shows the average of the means from 3 independent experiments ±SD. Statistical significance was determined using an unpaired t-test. **C**. RING1B^R70C^-LacR tethering does not cause repositioning of the array. (Top panel) Graphical illustration of the RING1B point mutation used in this study (Left panel) Heat map showing distribution of distance of the array from the nucleolus in 100 cells with mCherry-LacR, RING1B-LacR or RING1B^R70C^-LacR tethered arrays. RING1B-LacR differs significantly from the control as judged by a KS test (p value≤ 0.001). (Right panel) Mean nucleolar distance of the array in mCherry-LacR, RING1B-LacR and RING1B^R70C^-LacR tethered cells. The mean nucleolar distance of each replicate was calculated from measurements of nucleolar distance in 100 cells. The graph shows the average of the means from 3 independent experiments ±SD. Statistical significance was determined using an unpaired t-test. **D**. Tethering of RING1B causes deposition of a prominent H2A-K119 ubiquitin mark. H2A-K119 ubiquitin antibody staining in cells with tethered RING1B-LacR, RBX1-LacR or RING1B^R70C^-LacR. Scale bar: 5μm. The H2A-K119-ubiquitin signal was observed to colocalize with RING1B LacR in 70/100 cells. Colocalization was seen in 0/100 cells for mCherry-LacR, RBX1-LacR and RING1B^R70C^-LacR.

DDB2 is known to form various ubiquitin E3 ligase complexes that catalyze the ubiquitylation of histones and other DNA repair factors during NER [13, 14, 32, 34, 48]. Each complex harbors the DDB2-DDB1 heterodimer, while the cullins and the E3 ligases vary between the complexes. To establish whether the repositioning of the array requires the formation of a functional DDB2 complex, we tethered two naturally occurring point mutations of DDB2 to the array, DDB2^D307Y^ and DDB2^L350P^ (Figure 3A). These mutants lack the ability to bind DDB1 and were previously shown to abolish the formation of a functional E3 ligase complex (Supplementary Figure 3A) [23]. As opposed to DDB2-LacR, neither of the tethered DDB2 mutants caused re-localization of the array to the nucleolus. This implies that a functional E3 ligase complex is required for the re-localization of the array.

Given the localization phenotype of the DDB2 mutants (Figure 3A) we reasoned that the catalytic activity of the UV-DDB-CUL4 complexes or the UV-RING1B complex might form the basis for the repositioning of the array. To distinguish the E3 ligase complexes we tethered either RING1B-LacR (UV-RING1B complex) or RBX1-LacR (UV-DDB-CUL4A/4B complexes) to the array (Figure 3B). We noticed that only RING1B but not RBX1 repositioned the array to the proximity of the nucleolus (Figure 3B), even though both E3 ligases caused ubiquitylation of the array (Supplementary Figure 3B). Furthermore, we did not observe repositioning when binding an enzymatically inactive RING1B mutant (RING1B^R70C^-LacR) (Figure 3C) [49], although RING1B^R70C^ retained the ability to recruit DDB2-EGFP, similar to RBX1 and RING1B (Supplementary Figure 3C). Notably, while both RING1B and RBX1 caused ubiquitylation when tethered to the array (Supplementary Figure 3B), only RING1B generated a prominent H2A-K119 ubiquitin mark (Figure 3D). In order to further characterize the role of the UV-RING1B complex, we additionally measured the array decondensation upon RING1B tethering. It was shown previously, that the DDB2 dependent decondensation of the array does not depend on ubiquitylation [45]. In line with this previous finding, we also observed that tethering of RING1B does not lead to decondensation of the array (Supplementary Figure 3D).

**Figure 4:**
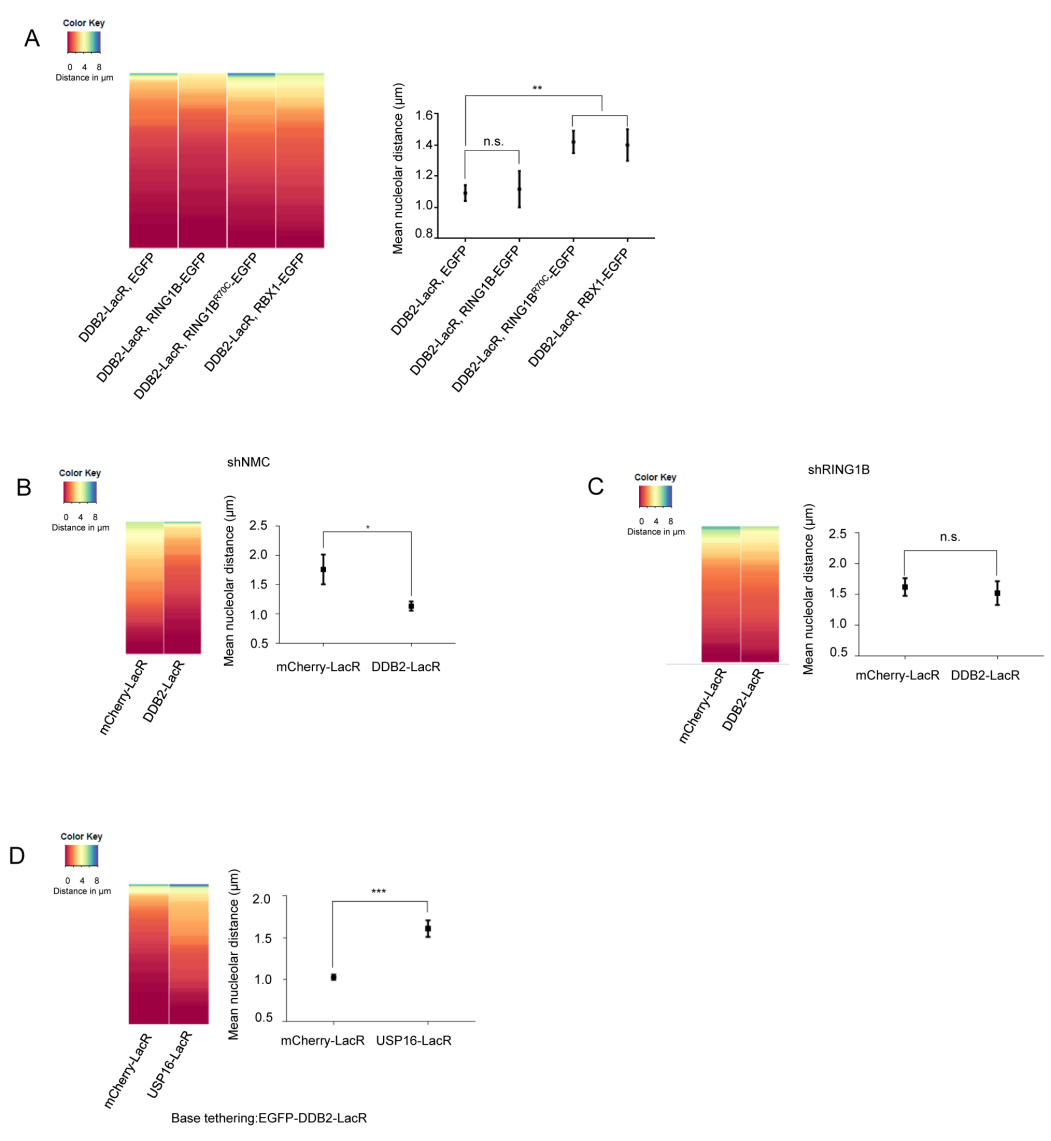
Repositioning of the array is dependent on the H2A-K119-ubiquitin mark set by RING1B **A**. DDB2-LacR mediated repositioning can be attenuated by competition between E3 ligases. (Left panel) Heat map showing distribution of nucleolar distance of the array in 100 cells. Cells with DDB2-LacR tethered arrays, expressing EGFP, RING1B-EGFP, RBX1-EGFP or RING1B^R70C^-EGFP were analyzed as shown. (Right panel) Mean nucleolar distance of the DDB2-LacR tethered arrays in cells expressing EGFP, RING1B-EGFP, RBX1-EGFP or RING1B^R70C^-EGFP as shown. The mean nucleolar distance of each replicate was calculated from measurements of nucleolar distance in 100 cells. The graph shows the average of the means from 3 independent experiments ±SD. Statistical significance was determined using an unpaired t-test. **B**., **C**. Knockdown of RING1B abolishes repositioning of a DDB2-LacR tethered array. (Left panel) Heat map showing distribution of the nucleolar distance of the array in 100 cells. mCherry-LacR or DDB2-LacR tethered arrays were analyzed in a control (shNMC) or RING1B knockdown (shRING1B) background. There was no significant difference between the control and DDB2-LacR tethered array in the shRING1B cells as judged by a KS test. (Right panel) Mean nucleolar distance of the control and DDB2-tethered array in shNMC or shRING1B cells. The mean nucleolar distance of each replicate was calculated from measurements of nucleolar distance in 100 cells. The graph shows the average of the means from 3 independent experiments ±SD. Statistical significance was determined using an unpaired t-test. **D**. Co-tethering of USP16-LacR abolishes repositioning of a DDB2-LacR tethered array. (Left panel) Heat map showing nucleolar distance of the array in 100 cells. EGFP-DDB2-LacR (base tethering) was co-tethered to the array along with either mCherry-LacR or mCherry-LacR-USP16. Co-tethering of USP16 leads to a significant repositioning of the array as judged by a KS test (p value≤ 0.0001). (Right panel) Mean nucleolar distance of the DDB2-LacR tethered array with mCherry-LacR and USP16 LacR co-tethering. The mean nucleolar distance of each replicate was calculated from measurements of nucleolar distance in 100 cells. The graph shows the average of the means from 3 independent experiments ±SD. Statistical significance was determined using an unpaired t-test.

Next, we performed experiments expressing RING1B (RING1B-EGFP), RBX1 (RBX1-EGFP) or the RING1B mutant (RING1B^R70C^-EGFP) in combination with DDB2-LacR in the U2OS 2-6-3 cell line. We observed recruitment of all EGFP tagged E3 ligases to the DDB2-LacR array, further confirming the formation of the respective E3 ligase complexes (Supplementary Figure 4A). Expression of RING1B-EGFP did not significantly change the localization observed with tethered DDB2-LacR alone (Figure 4A). However, simultaneous expression of RING1B^R70C^-EGFP or RBX1-EGFP caused an increase in the mean distance from the nucleolus suggesting they might compete with functional endogenous RING1B for binding DDB2-LacR thereby precluding the generation of the UV-RING1B complex. Next we assessed whether RING1B depletion affected the repositioning of the array. Depletion of RING1B (shRING1B) in the U2OS 2-6-3 cell line caused a reduction of DDB2 mediated re-localization (Figure 4C and Supplementary Figure 4D) when compared to control cells (shNMC) (Figure 4B). Finally, we wanted to confirm that the loss of the repositioning occurs due to absence of H2A-ubiquitylation, and not due to absence of functional RING1B. Hence, we performed a double tethering experiment with the H2A-ubiquitin specific deubiquitinase USP16 [50]. Simultaneous tethering of RING1B and USP16 to the array completely eliminated the H2A-K119 ubiquitin mark from the array (Supplementary Figure 4B and 4C). Importantly, simultaneous tethering of USP16 and DDB2 to the array provoked a total loss of the re-localization observed for DDB2 alone (Figure 4D). Thus, our data suggest that absence of H2A-K119-ubiquitylation prevents the re-localization of the array to the nucleolus in spite of presence of a functional E3 ligase complex.

### ZRF1 is present in the nucleolus and facilitates relocalization of chromatin

**Figure 5:**
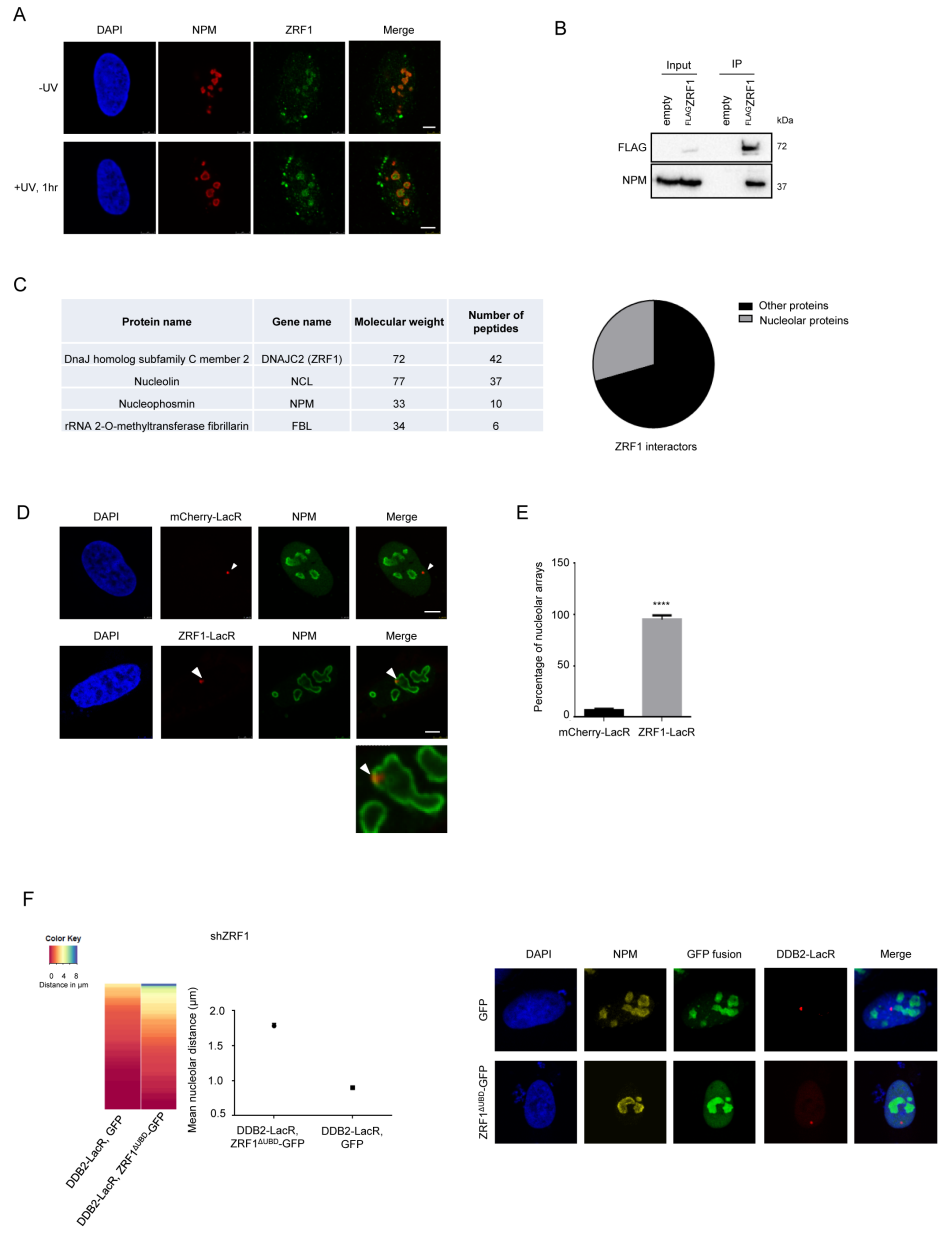
ZRF1 is present in the nucleolus and causes relocalization of chromatin **A**. ZRF1 localizes to nucleoli. Immunofluorescence images showing intra-nuclear distribution of ZRF1 in pre-extracted control cells and cells exposed to UV irradiation. Nucleoli are marked by Nucleophosmin (NPM). Pre-extraction washes off unbound nuclear and cytoplasmic proteins and enables visualization of chromatin-associated proteins. Scale bar: 5μm. **B**. ZRF1 interacts with NPM. Purifications from HEK293T cells transfected with either empty vector or ^FLAG^ZRF1, show specific interaction of NPM with ZRF1 **C**. ZRF1 interacts with major components of the nucleolus. ^FLAG^ZRF1, along with its interactors, was purified from HEK293T cells and the purified material was subjected to mass spectrometry. Multiple components of the nucleolus were found to interact with ZRF1. (Left panel) the table shows peptide numbers for selected proteins in the FLAG purification. (Right panel) 30% of all interacting proteins were found to be nucleolar, as assessed by comparison with the nucleolar protein database NOPdb (https://www.ncbi.nlm.nih.gov/pmc/articles/PMC1347367/#!po=8.62069). **D**. Tethering of ZRF1 to a LacO array leads to repositioning of the array. Representative images of mCherry-LacR-ZRF1 or mCherry-LacR tethered LacO arrays. Nucleoli are marked by Nucleophosmin (NPM). Scale bar -5μm **E**. Quantification of nucleolar arrays (distance from nucleolus = 0μm) in control (mCherry-LacR) and ZRF1 (ZRF1-LacR) tethered arrays. 90-100 cells were counted per experiment. The graph shows the mean ±SD. (*n* = 3). Significance was determined by an unpaired t-test. **F**. Overexpression of ZRF1^ΔUBD^-GFP abolishes repositioning of a DDB2-LacR tethered array. (Left panel) Heat map showing distribution of the nucleolar distance of the array in 100 cells. DDB2-LacR tethered arrays were analyzed in a ZRF1 knockdown (shZRF1) background, after expression of either nucleolar GFP or nucleolar ZRF1^ΔUBD^-GFP. There was a significant difference between the DDB2-LacR tethered array in the control and mutant overexpression cells as judged by a KS test. (Middle panel) Mean nucleolar distance of the DDB2-tethered array in the corresponding cells. The mean nucleolar distance of each replicate was calculated from measurements of nucleolar distance in 100 cells. The graph shows the average of the means from 3 independent experiments ±SD. Statistical significance was determined using an unpaired t-test. (Right panel) Immunofluorescence images showing cells expressing nucleolar targeted GFP or ZRF1^ΔUBD^-GFP, along with DDB2-LacR. Nucleoli are marked by NPM and nuclei with DAPI. **G**. Knockdown of XPC abolishes repositioning of a DDB2-LacR tethered array. (Left panel) Heat map showing distribution of the nucleolar distance of the array in 100 cells. mCherry-LacR or DDB2-LacR tethered arrays were analyzed in a XPC knockdown (shXPC) background. There was no significant difference between the control and DDB2-LacR tethered array in the shXPC cells as judged by a KS test. (Right panel) Mean nucleolar distance of the control and DDB2-tethered array in shXPC cells. The mean nucleolar distance of each replicate was calculated from measurements of nucleolar distance in 100 cells. The graph shows the average of the means from 3 independent experiments ±SD. Statistical significance was determined using an unpaired t-test. (G) Hypothetical model for nucleolar tethering of damaged DNA via H2A K119-ubiquitylation.

As the UV-RING1B complex is required for re-localization of the array to the vicinity of the nucleolus, we next asked how the DNA is anchored at the nucleolus for repair. ZRF1, a reader of the H2A-ubiquitin mark at lysine 119, was recently shown to be essential for NER. ZRF1 requires both H2A-ubiquitylation and XPC to bind to damaged chromatin [34]. Investigating the nuclear distribution of ZRF1 after pre-extraction of U2OS cells, we detected ZRF1 in the nucleolus as seen by simultaneous Nucleophosmin (NPM) staining (Figure 5A). Similarly, EGFP tagged ZRF1 showed nucleolar localization (Supplementary Figure 5A). In agreement with these findings we detected NPM in immunoprecipitations of endogenous ZRF1 as well as ectopically expressed ZRF1^FLAG^ (Figure 5B, Supplementary Figure 5B). Furthermore, immunoprecipitations of FLAG-tagged ZRF1 followed by mass spectroscopy indicated that it associates with many nucleolar proteins, including Nucleophosmin, Fibrillarin and Nucleolin (Figure 5C and Supplementary Table 1). We did not detect any major changes in the interaction partners of ZRF1 in control versus irradiated cells (Supplementary Figure 1C and Supplementary Table 1), suggesting that the bulk of ZRF1 is constitutively present in the nucleolus. Next, we explored if chromatin-associated ZRF1 also localized to the nucleolus. Upon tethering ZRF1, the LacO array showed a nucleolar positioning in ≈ 90 % of the analyzed cells (Figure 5D and 5E). Importantly, tethered mCherry-LacR-ZRF1 was able to bind XPC-GFP, in agreement with our recent findings [34] thus confirming functional activity of tethered ZRF1 (Supplementary Figure 5D). Collectively these data show that a randomly distributed DNA locus is repositioned to the nucleolus upon ZRF1 binding.

We next assessed if loss of ZRF1 affected the nucleolar positioning of a DDB2 tethered array. To this end we created ZRF1 U2OS 2-6-3 knockdown cell lines (Supplementary Figure 5F). However, when analyzing the knockdown cells, we observed only insufficient depletion of nucleolar ZRF1 whereas the cytoplasmic protein levels were reduced significantly (data not shown). In order to circumvent the lack of sufficient knockdown of nucleolar ZRF1, we selectively targeted a ZRF1 deletion mutant, lacking the ubiquitin binding domain (ZRF1^ΔUBD^), to the nucleolus of ZRF1 knockdown cells [51, 52] (Supplementary Figure 5E). Upon expressing the deletion mutant specifically in the nucleolus, we completely lost the DDB2 mediated repositioning of the array (Figure 5F). Additionally, we analyzed the repositioning of the array after knockdown of XPC. In absence of XPC, ZRF1 cannot bind chromatin even in presence of H2A-ubiquitylation [34]. Depletion of XPC resulted in a complete loss of the re-localization phenotype when utilizing a DDB2-tethered array (Figure 5F and Supplementary Figure 5E). Collectively, these data suggest that ZRF1 anchors damaged DNA to the nucleolus and that it presumably interacts with H2A-ubiquitin at the damage site via its Ubiquitin-binding domain in a XPC dependent manner.

## DISCUSSION

NER is one of the most prominent DNA repair pathways. In order to investigate if NER occurs in defined repair centers, we used a modified UDS protocol to look at the nuclear distribution of repair occurring at various time points after UV irradiation. We observed appearance of specific repair foci starting from 8 hours and lasting up to 24 hours after UV exposure, which overlapped with nucleoli. Upon quantification, we observed that repair in the nucleolus occurs at all the measured time points (0- 24 hours), however at later time points the majority of the repair consists of nucleolar repair (Figure 1A). These relatively late time points of DNA repair are in agreement with the timing observed for the removal of CPDs, specifically from heterochromatin in a DDB2 dependent manner (Bykov et al., 1999; Zhao et al., 2002; Han et al. 2016). We also observed translocation of the repaired DNA out of the nucleolus at both early and late time points (Figure 1B), implying that it is non-nucleolar DNA that is actively brought to the nucleolus for repair. In addition, we observed repair activity in the nucleolus upon irradiation through a micropore membrane, demonstrating migration of the damaged DNA towards the nucleolus (Figure 1E). We found accumulation of XPA in the nucleolus very early after UV damage, which implies the presence of a functional repair machinery. Interestingly, loss of NPM causes reduced NER (Wu et al., 2002a) whereas its overexpression increases survival and NER capacity (Wu et al., 2002b) supporting a role for the nucleolus in NER. In line with these observations, our data suggest that the nucleolus might facilitate NER by organizing repair by tethering of damaged chromatin (Figure 5G).

In order to determine the mechanism of translocation of damaged DNA to the nucleolus for repair, we used a tethering system to mimic a lesion [23, 53]. Tethering two subunits of the UV-RING1B complex (DDB2 or RING1B) we observed a re-localization of the array close to the nucleolus (mean ≈1μm). Experiments in different systems such as flies, yeast as well as mammalian cells have demonstrated that chromatin undergoes rapid motion in a radius of 0.5-1μm (Chubb et al., 2002; Heun et al., 2001; Marshall et al., 1997; Vazquez et al., 2001) making our repositioning functionally relevant. RING1B is most widely known as a component of PRC1 and for its role in gene silencing during development. RING1B specifically catalyzes ubiquitylation of H2A at lysine 119. Here, we show that during NER, H2A-K119 ubiquitylation plays a definite role in tethering damaged chromatin to the nucleolus. This tethering requires ZRF1 as the nucleolar anchor, and occurs in an XPC dependent manner. We have shown previously that ubiquitylation at H2A-K119 precedes RBX1-mediated ubiquitylation [34] thereby initiating a ubiquitin signaling cascade. Since ZRF1 is responsible for exchanging the E3 ligases at the damage site [34], it is likely that this remodeling occurs at the nucleolus, too. Recently it was shown that many of the PRC1 related functions of RING1B are independent of its E3 ligase activity (Eskeland et al., 2010; Illingworth et al., 2015). However, its function in NER requires functional catalytic activity. Thus, our data propose a novel function for RING1B catalyzed H2A-ubiquitylation in nuclear chromatin organization (Figure 5G).

ZRF1 is one of the few known readers of H2A-K119 ubiquitin. ZRF1 function has been shown to actively displace RING1B not only in NER but also in the course of cellular differentiation. Upon differentiation of cells, ZRF1 is present at the promoters of many developmental genes such as HOX genes. In this study, we show that tethering ZRF1 to chromatin relocates the chromatin to the nucleolus. This finding creates an interesting repercussion for ZRF1-mediated transcriptional activation. We speculate that the activation of developmental loci by ZRF1 might also potentially involve their migration to the nucleolus.

The nucleolus, along with the nuclear lamina, is considered an important nuclear structure that binds specific genomic regions and plays a major role in the three-dimensional organization of the genome (Lemaitre and Bickmore, 2015; Nemeth et al., 2010). In addition, about half of the nucleolar proteome consists of proteins involved in functions other than ribosome biogenesis, viz regulation of tumor suppressor and proto-oncogene activities, cell-cycle control, DNA replication, DNA repair, and stress signaling (Boisvert et al., 2007; Boulon et al., 2010; Koehler and Hanawalt, 1996). A potential function of re-localizing chromatin to the nucleolus may be to aid in chromatin remodeling. Ribosome biogenesis and chromatin remodeling, both involve regulation of nucleic acid-protein interactions, and the nucleolus contains much of the machinery required for this process. Notably, Nucleophosmin functions as a histone chaperone and as a sink for histones (Lindstrom, 2011).

In conclusion, we propose a novel role for RING1B mediated H2A-ubiquitylation in sub-nuclear localization of NER. ZRF1, in combination with XPC, tethers the damaged chromatin to the nucleolus and facilitates repair. It remains to be seen which fraction of NER occurs in the nucleolus, and how the decision is made for a lesion to be repaired there. Another interesting open question is whether the recognition and tethering of chromatin bearing the H2A-ubiquitylation mark by ZRF1 is a central mechanism playing a role in other cellular processes.

## MATERIALS AND METHODS

### Cell culture and cell lines

HEK293T, U2OS and U2OS 2-6-3 cells were cultured in DMEM supplemented with 10% FBS at 37°C and 5% CO2. U2OS 2-6-3 medium was additionally supplemented with 100 μg/ml Hygromycin to maintain stable insertion of the LacO cassette. Normal skin fibroblasts (GM15876) were purchased from the Coriell Cell Repositories and cultured in DMEM, supplemented with 15% FBS.

Transfection of U2OS 2-6-3 and HEK293T cells was performed by Lipofectamine 2000 (Invitrogen) transfection according to manufacturer's instructions. Information on the plasmids used in this study is provided in the supplementary information.

### Lentiviral transduction

Gene knockdown in U2OS 2-6-3 cells was performed by introduction of MISSION pLKO.1-shRNA plasmids (Sigma-Aldrich) targeting the respective gene using the 3rd generation lentivirus system. Plasmids contained the following sequences (Sigma): non-mammalian control (NMC) (TRC1/1.5), RING1B (TRCN0000033697), XPC (TRCN0000307193), ZRF1 (TRCN0000254058).

### Immunofluorescence and drug treatments

Cells were fixed in 4%PFA for 10 min at room temperature. Pre-extraction with CSK buffer (10mM PIPES pH- 7.4, 100mM NaCl, 300mM sucrose, 3mM MgCl_2_) containing 0.2% Triton-X, for 5 min on ice, was performed prior to fixation when indicated. Cells were incubated overnight with primary antibody at 4 °C. Subsequently cells were incubated with Alexa-fluorophore-conjugated secondary antibodies (Life Technologies). The mounting was carried out in Vectashield with DAPI (Vector Laboratories).

Micropore irradiation experiments were performed on MRC5 fibroblasts. Cells were exposed to localized UV damage (100 J/m2) using a micropore membrane with 5-μm pore size as described previously (Katsumi et al., 2001).

PARP inhibitor treatment was performed as described in (Luijsterburg et al. 2012b). Briefly, 5mM IPTG was added to the cells before and during transfection with the mCherry-LacR-fusion plasmid. IPTG was washed out 24 hours post transfection, and replaced with medium containing 1 μM PARP inhibitor (KU-0058948). Cells were incubated with inhibitor for 16 hours, followed by fixation and staining.

### Measurement and statistical analysis of array distributions

For tethering experiments, nucleolar distances were measured in 100 randomly picked transfected cells per sample. All cells with visible tethered array were used, consisting of all levels of LacR-fusion protein expression. Nucleolar distance was measured as the distance of the array, to the closest NPM marked nucleolus. Distributions of nucleolar distances for control and test sample were compared by the Kolmogorov-Smirnov (KS) test. For each experiment, mean nucleolar distances were calculated from a minimum of three independent experiments, each showing a significant difference in distributions as indicated by the KS test. The respective means were then compared by an unpaired t-test. All statistical tests were performed using GraphPad Prism.

### UDS experiments

UDS experiments were performed as described elsewhere [54]. Briefly, fibroblasts were serum starved for 24 hours, irradiated with UV light (20J/m^2^) and incubated with 10μM EdU (Thermo Fisher) for 2 hours at the indicated time point after UV exposure. At this stage, the cells were then fixed and further processed to visualize localization of repaired DNA. In experiments tracking re-distribution of repaired DNA, medium was replaced by EdU free medium, and repair was allowed to proceed till 24 hours post UV. Cells were then fixed and processed for further staining. Alexa-555-azide (Thermo Fisher) was conjugated to EdU using the click-reaction. The cells were additionally incubated with Nucleophosmin antibodies, followed by Alexa-488 secondaries (anti-mouse) before mounting in Vectashield with DAPI.

### Microscopy and image analysis

Images were acquired with the LAS AF software (Leica) using a TCS SP5 confocal microscope (Leica) with a 63x/1.4 oil immersion objective. The following lasers were used: 50 mW UV diode (405 nm), 65 mW argon, 20 mW DPSS (561 nm) and 10 mW HeNe (633 nm).

Analysis of nuclear distribution of UDS was carried out by a self-written Fiji/ImageJ macro. Briefly, single z-planes with nucleoli in focus were captured for each cell. Single channel fluorescence images (NPM-488, DAPI) were smoothed, threshholded and converted to binary masks. DAPI was used as a nuclear mask, while NPM was used as a nucleolar mask and a combination of the two (nuclear-nucleolar) served as the nucleoplasmic mask. The binary masks were used to measure the mean intensity for the nucleus, nucleolus, and nucleoplasm in the corresponding EdU image. These values were used to calculate the Nucleolar Repair Index as (Nucleolus^mean^- Nucleoplasm^mean)^/Nucleus^mean^ ×100. NRI was calculated for all the nucleoli from ≈ 100 cells per time point.

### FLAG purifications

Cells were UV irradiated (20J/m^2^) and harvested 1 hour after exposure (unless stated otherwise). FLAG affinity purifications were performed using FLAG-M2 agarose beads as already published [51].

### Mass spectrometry

Mass-spectrometry sample preparation, measurement and database search were performed as described elsewhere [55]. Gradient lengths of 45 or 105 min were chosen depending on the IP material obtained. Raw files were processed with MaxQuant (version 1.5.2.8) and searched against the *Homo sapiens* Uniprot database (25. February 2012) using the Andromeda search engine integrated into MaxQuant and default settings were applied. Proteins with at least 2 peptides, one of them unique, count as identified.

### Purification of nucleoli

Nucleolar purification was performed as previously described [56] with slight modifications. Roche's complete Protease Inhibitor Cocktail (PI) was added to all solutions and the entire protocol was performed on ice. Briefly, cells were scraped off in Solution I (0.5M Sucrose, 3mM MgCl_2_ +PI), chilled to -20 °C to quench metabolic activities, pelleted, and washed once more with Solution I. Washed cells were resuspended in 1ml Solution I and sonicated for 7 cycles (10s on/10s off) in a Bioruptor (Diagenode). Sonicated cells were checked under a microscope to ensure efficient cell lysis. Cell lysate was then layered over 1.4 ml of Solution II (1M Sucrose, 3mM Mg Cl_2_ +PI) and centrifuged at 1800g for 10 min at 4 °C. The supernatant was carefully removed and the nucleolar pellet was resuspended in Laemmli buffer and boiled, or subjected to subsequent salt extraction.

### Nucleolar fractionation

Purified nucleoli were incubated with Salt Extraction buffer I (50mM Tris pH 7.5, 0,05%NP40, 0.1M NaCl + PI) for 10 min on ice. Nucleoli were centrifuged at 2800g for 5 min and the supernatant was saved as the 0.1M salt soluble fraction. Similar extractions were performed sequentially with Salt Extraction buffer II (50mM Tris pH 7.5, 0,05%NP40, 0.2M NaCl + PI) and Salt Extraction Buffer III (50mM Tris pH 7.5, 0,05%NP40, 0.3M NaCl + PI). After the last extraction the remaining pellet was saved as chromatin bound fraction. Laemmli buffer was added to the samples and they were boiled for 10 min. Subsequently the samples were analysed by SDS-PAGE and western blotting. Histones were found to be prominently in the insoluble fraction, thus verifying that it represents the chromatin bound fraction.

### UV irradiation

Cells were irradiated with 20J/m^2^ UV-C using a CL-1000 UV-crosslinker (UVP) unless stated otherwise.

### Plasmids and antibodies

mCherry-LacR-DDB2, mCherry-LacR-DDB2^D307Y^, mCherry-LacR-DDB2^L350P^, EGFP-DDB2, EGFP- DDB2^D307Y^, EGFP- DDB2^L350P^ were kindly provided by Nico Dantuma. XPC-GFP and NLS-R7-GFP was gifted by Cristina Cardoso. GBP-LacR was obtained from Heinrich Leonhardt. mCherry-LacR-nostop was a gift from Vassilis Roukos.

mCherry-LacR-RING1B, mCherry-LacR-CSA, mCherry-LacR-RBX1, mCherry-LacR-RING1B^R70C^, mCherry-LacR-ZRF1, mCherry-TetR-USP16, EGFP-LacR-RING1B, EGFP-LacR-DDB2, EGFP-ZRF1, EGFP-NLS-ZRF1, NLS-R7-GFP-ZRF1^ΔUBD^, EGFP-RING1B, EGFP-RING1B^R70C^, EGFP-RBX1, FLAG-ZRF1 were cloned. For details please contact the authors.

Antibodies used in this study were: XPA (Genetech), XPC (Abcam), DDB2 (MyBioSource), XPB (Santa Cruz Biotechnology), Fibrillarin (Thermo Scientific), ZRF1 (Novus Biologicals), Nucleophosmin (Abcam ab10530), DDB1 (Bethyl), Ubiquitin (clone P4D1, Cell Signalling).

## SUPPLEMENTARY MATERIALS FIGURES AND TABLES




