# Supplementary material for: Nuclear organization of nucleotide excision repair is mediated by RING1B dependent H2A-ubiquitylation
Source: Oncotarget. 2017 Mar 11;8(19):30870–87. doi: 10.18632/oncotarget.16142 (PMC5458174; doi:10.18632/oncotarget.16142)
Supplement: Supplementary file 1 [file oncotarget-08-30870-s001.pdf]

Nuclear organization of nucleotide excision repair is mediated by RING1B dependent H2A-ubiquitylation

Supplementary Material

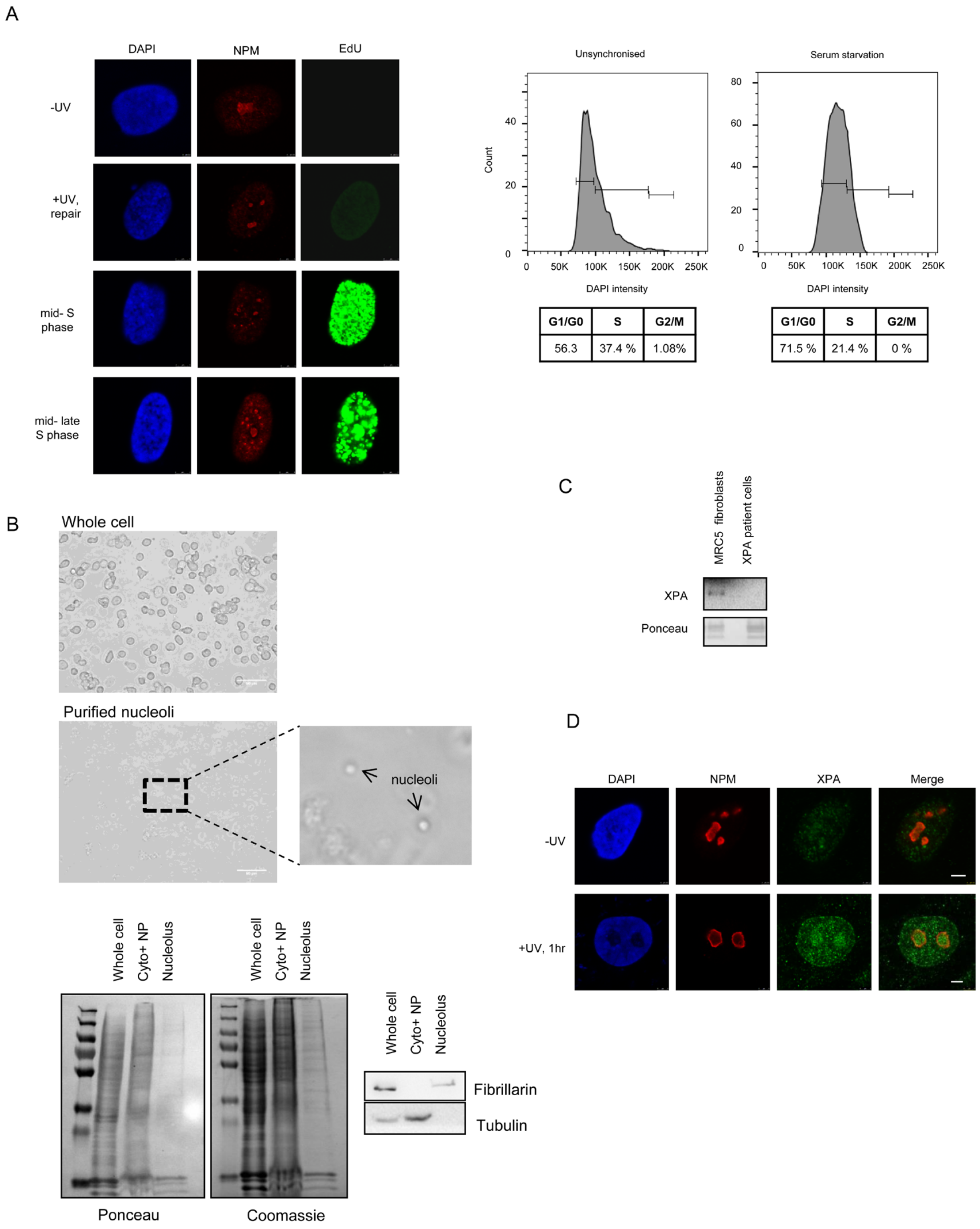

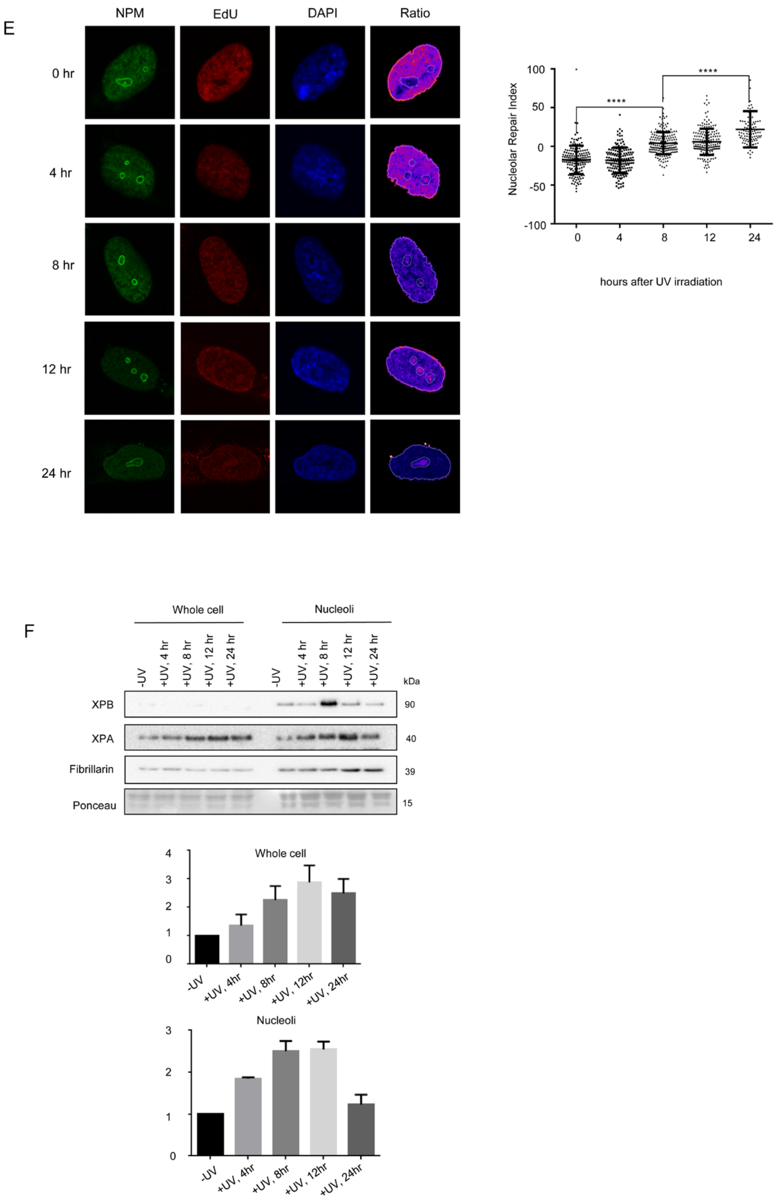

Figure S1: NER is routed to the nucleolus. (a) (Left panel) Immunofluorescence images showing EdU incorporation (green) in control cells unexposed to UV (-UV), non-S phase cells showing post-damage repair activity (+UV, repair) and S phase cells. Nuclei are demarcated by DAPI staining, and nucleoli marked by Nucleophosmin (NPM). (Right panel) FACS profiles for unsynchronized normal human fibroblasts (untransformed), and after 24 hour serum starvation. The tables show percentage of the cells in G0/G1, S and G2/M phase, respectively. (b) Assessment of purity of nucleolar fractions. (Top panel) bright field image showing nucleoli in cell lysate. (Bottom panel) Coomassie staining of extracts from: whole cells, cytoplasmic and nucleoplasmic fraction (Cyto+NP) and nucleolar fraction. Western blots showing the purity of nucleolar fractions. Fibrillarin was used as a marker for the nucleolus and Tubulin as a marker for the Cyto+NP fraction. (c) Western blot images to show specificity of XPA antibody. Cell extracts from XPA deficient fibroblasts (XPA patient cells) and MRC5 fibroblasts were loaded on a gel and blotted for XPA. (d) XPA accumulates in the nucleolus post UV exposure. Immunofluorescence images showing staining of NPM and XPA in pre-extracted MRC5 cells unexposed to UV, or 1 hour after exposure to a 20J/m^2 UV dose. (e) (Left panel) Representative images of active repair foci indicated by EdU incorporation, at the indicated time points post UV exposure. The figure additionally shows the image generated after normalization of the EdU signal with respect to DAPI signal (Ratio). Cells are co-stained with NPM to show nucleolar overlap of repair foci. Scale bar: 5μm. (Right panel; Figure 1B) The graph shows the Nucleolar Repair Index (NRI) values of nucleoli from ≈100 non-S phase cells at the indicated time points post UV exposure. NRI values were calculated with respect to the normalized EdU image (Ratio). Statistical significance was calculated using a Mann-Whitney test. (f) XPA is enriched in the nucleolus post UV exposure. (Top panel) Western blots showing levels of the indicated proteins in either whole cell extracts, or nucleolar fractions at the stated time points after UV exposure. (Bottom panels) The XPA band intensity was normalized to Fibrillarin as a loading control, and the normalized band intensity was plotted. The graph shows the mean±SEM, n=3.

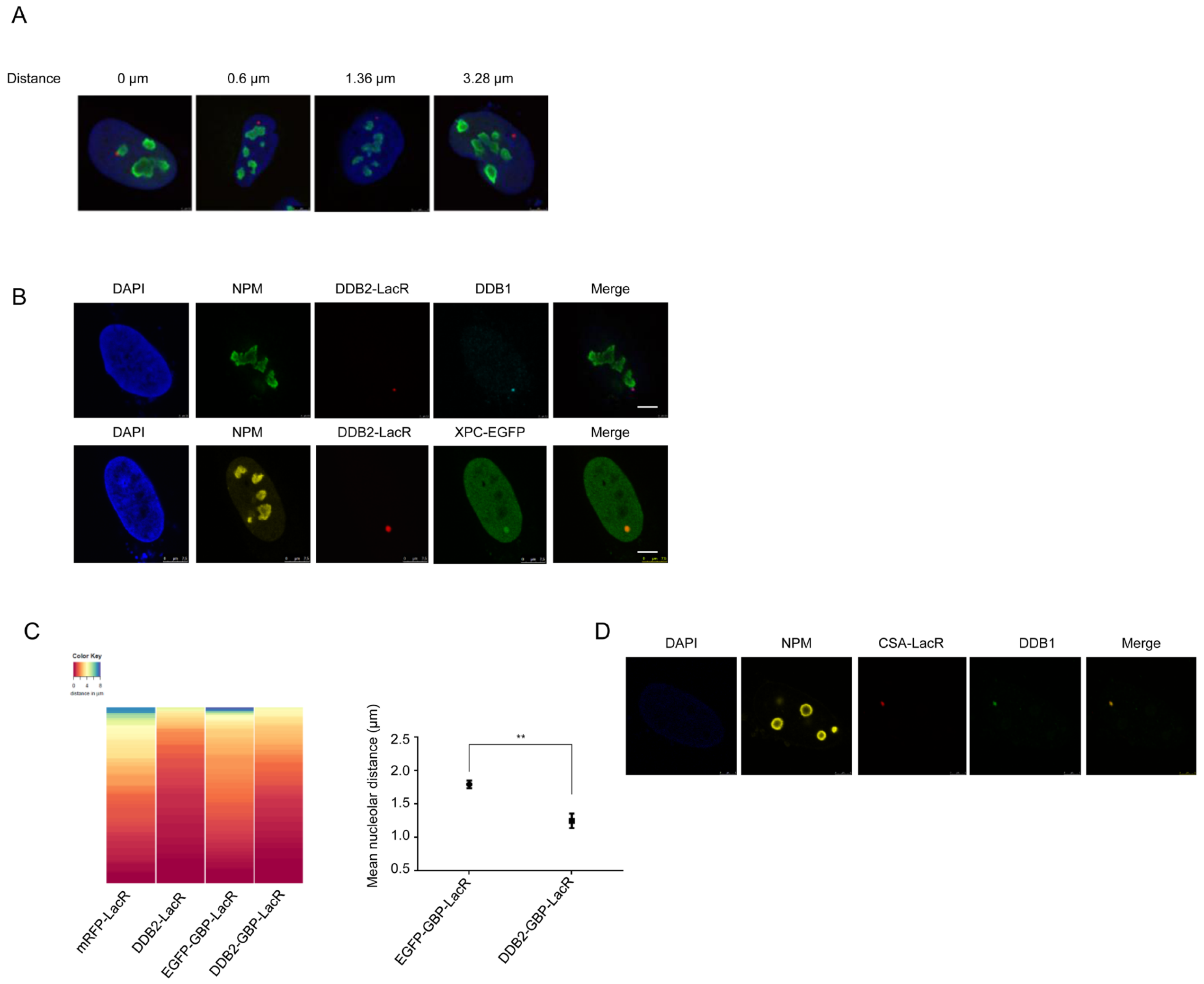

Figure S2: Tethering of DDB2 leads to relocalization of the LacO array. (a) Representative immunofluorescence images showing the position of a LacO array with respect to the nucleolus. The corresponding nucleolar distance is indicated. Nucleoli are marked by NPM. Scale bar: 5 µm. (b) Tethered DDB2-LacR shows co-localization with endogenous DDB1 and XPC-EGFP. Immunofluorescence images showing the recruitment of endogenous DDB1 or XPC-GFP to an mCherry-LacR-DDB2 tethered array. Nucleoli are marked by NPM. Scale bar: 5 µm. (c) DDB2 shows a similar effect on the array distribution when tethered indirectly. (Left panel) Heat map showing the distribution of the nucleolar distance of the array in 100 cells expressing either EGFP or DDB2-EGFP along with GFP-binding protein (GBP)-LacR. DDB2-EGFP tethering leads to a significant difference in distribution of the array when compared to EGFP tethering as judged by a KS test. (p value≤ 0.0001). (Right panel) Mean nucleolar distance of the array in EGFP-GBP-LacR and DDB2-GBP-LacR arrays. The mean nucleolar distance of each replicate was calculated from measurements of nucleolar distance in 100 cells. The graph shows the average of the means from 3 independent experiments ±SD. Statistical significance was determined using an unpaired t-test. (d) Tethered CSA-LacR interacts with DDB1. Immunofluorescence image showing colocalization of tethered CSA-LacR with endogenous DDB1. Scale bar: 5 µm.

A

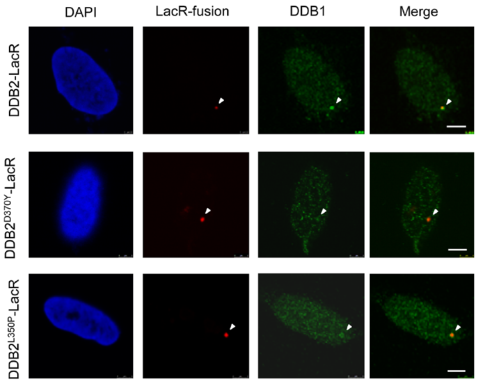
DAPI
LacR-fusion
DDB1
Merge
DDB2-LacR
DDB2^{D370Y}-LacR
DDB2^{L350P}-LacR

B

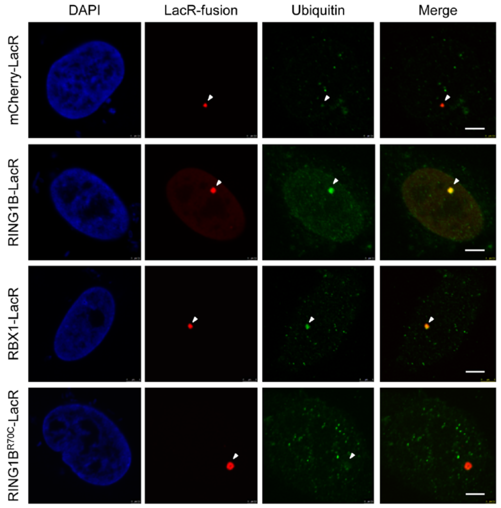
DAPI
LacR-fusion
Ubiquitin
Merge
mCherry-LacR
RING1B-LacR
RBX1-LacR
RING1B^{R70C}-LacR

C

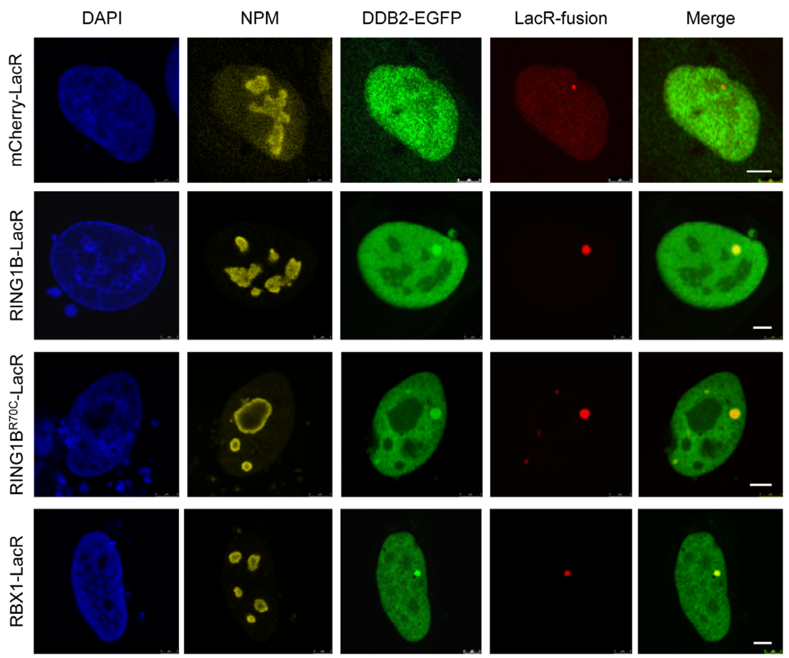

D

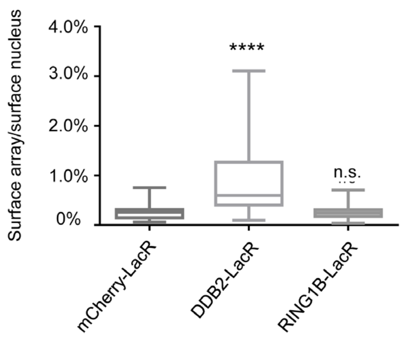

Figure S3: UV-DDB E3 ligase complexes are formed at the LacO array.

(a) Immunofluorescence images showing recruitment of endogenous DDB1 to tethered DDB2-LacR, DDB2^{D307Y}-LacR and DDB2^{L350P}-LacR tethered arrays. Scale bar: 5 µm. (b) Tethered RING1B and RBX1 facilitate ubiquitylation at the array. Immunofluorescence images showing accumulation of ubiquitin at the array tethered with the respective proteins. The observed co-localization was seen in 50/50 cells imaged for all conditions. Scale bar: 5 µm. (c) Tethered RING1B, RING1B^{R70C} and RBX1 all show recruitment and binding of DDB2-EGFP. Immunofluorescence images showing co-localization of DDB2-EGFP with the respective tethered proteins. Nucleoli are marked by NPM. Colocalization was assessed in cells showing comparable expression levels of the EGFP and the LacR fusion proteins. Colocalization was observed in 0/30 cells for mCherry-LacR, 30/30 cells for RBX1-LacR, 26/30 cells for RING1B^{R70C}-LacR and 27/30 cells for RING1B-LacR. Scale bar- 5 µm (d) Tethering of RING1B-LacR does not lead to array decondensation. The graph shows the distribution (Min to Max) of the percentage of nuclear area occupied by the specified tethered array. Array size was measured in 50-100 cells from two independent experiments. Statistical significance was assessed by an unpaired t-test.

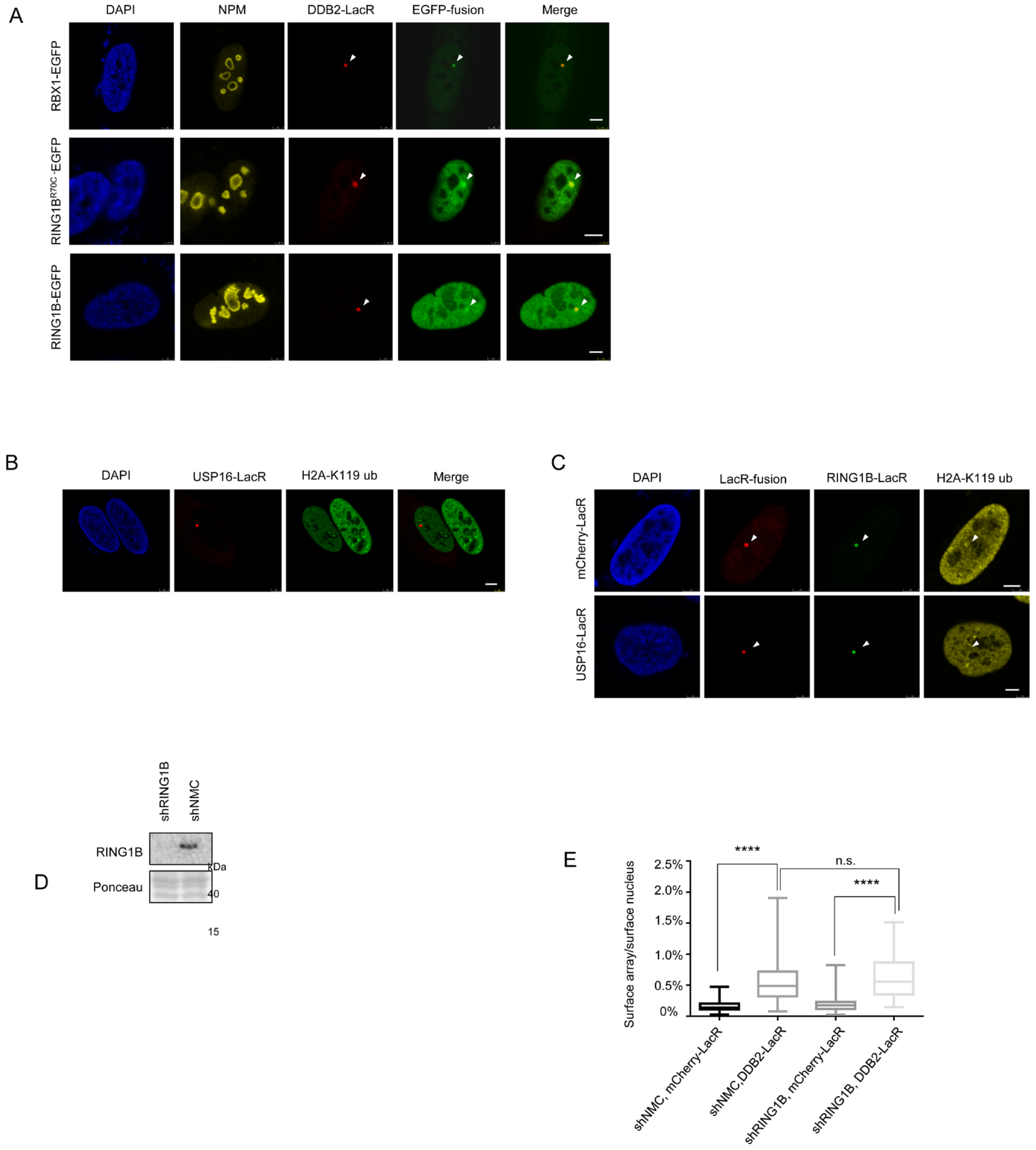

Figure S4: RING1B mediated H2A-ubiquitylation is required for relocation of array

(a) Tethered DDB2 shows recruitment of RING1B-EGFP, RING1B^{R70C}-EGFP and RBX1-EGFP. Immunofluorescence images showing recruitment of the respective EGFP tagged proteins to a DDB2-LacR tethered array. Nucleoli are marked by NPM. Colocalization was assessed in cells showing comparable expression levels of the EGFP and the LacR fusion proteins. Colocalization was observed in 30/30 cells for RBX1-EGFP, 25/30 cells for RING1B^{R70C} and 27/30 cells for RING1B-EGFP. Scale bar: 5 µm. (b) Expression of USP16-LacR leads to overall loss of H2A-K119 ubiquitylation. Immunofluorescence images showing reduced H2A-K119 ubiquitin levels both, at the array, as well as in the entire nucleus of cells expressing mCherry-USP16-LacR. Absence of H2A-K119 signal at the tethered USP16-LacR array was observed in 50/50 cells. Scale bar: 5 µm. (c) Co-tethering of USP16-LacR leads to loss of the RING1B-LacR catalyzed H2A K119 mark. Immunofluorescence images showing the distribution of H2A-K119 ubiquitylation after co-tethering of EGFP-LacR-RING1B with either mCherry-LacR or mCherry-LacR-USP16. Absence of H2A-K119 signal at the double tethered RING1B-LacR, USP16-LacR array was observed in 50/50 cells. Scale bar: 5 µm. (d) Western blot showing knockdown of RING1B in shRING1B cells compared to control (shNMC). (e) Knockdown of RING1B does not affect DDB2 induced array decondensation. The graph shows the distribution (Min to Max) of the percentage of nuclear area occupied by the specified tethered arrays in a control (shNMC) and RING1B knockdown (shRING1B) background. Array size was measured in 50-100 cells from two independent experiments. Statistical significance

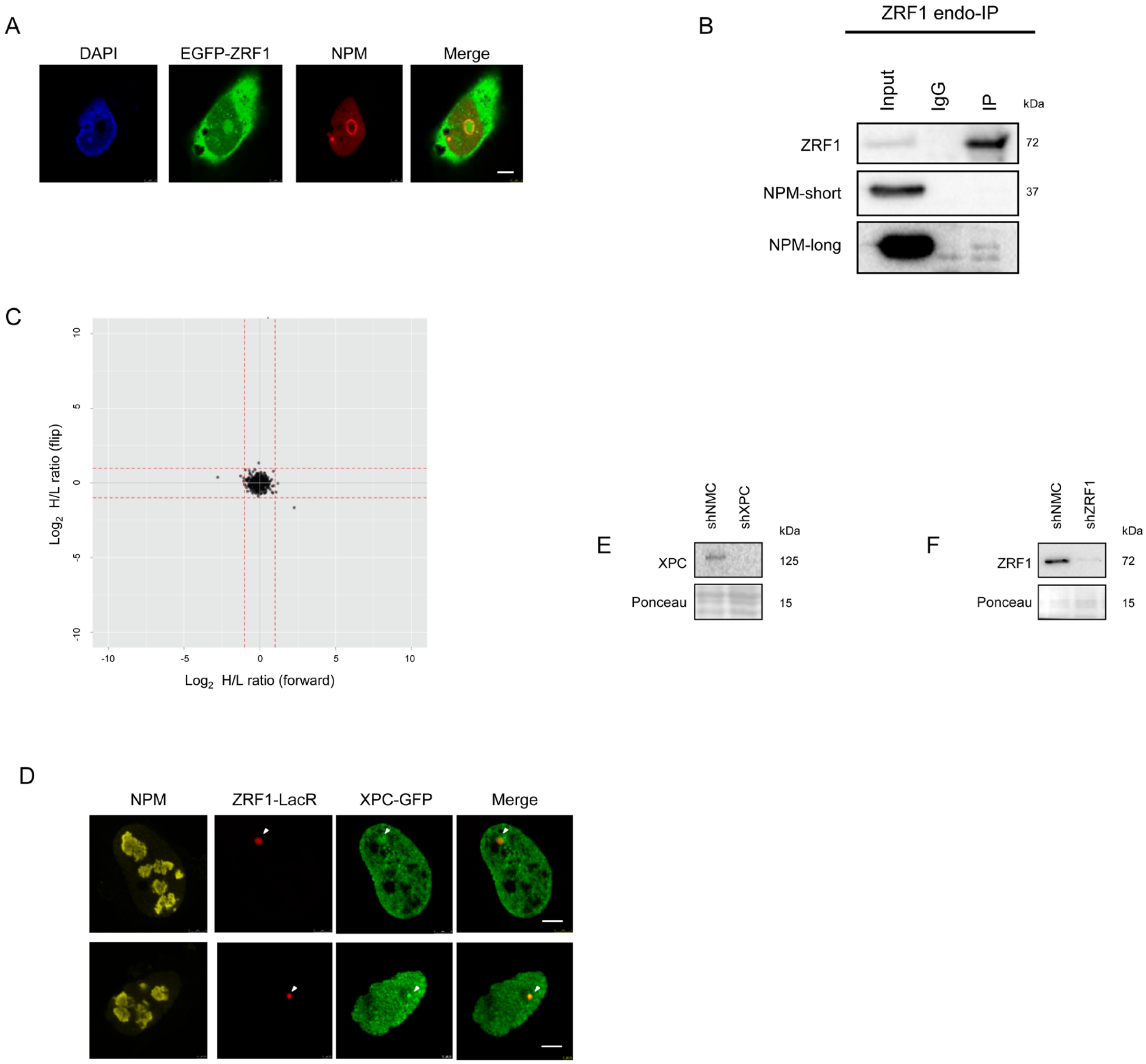

Figure S5. Nucleolar functions of ZRF1. (a) The distribution of EGFP tagged ZRF1 matches the distribution of endogenous ZRF1. Immunofluorescence images showing intracellular localization of EGFP-ZRF1 in non pre-extracted cells. Nucleoli are marked by NPM staining. Scale bar: 5 µm. (b) Endogenous ZRF1 interacts with NPM. Immunoprecipitation of endogenous ZRF1 showed co-immunoprecipitation of NPM (c) Scatterplots depicting SILAC heavy/light (H/L) ratios for proteins identified in both the “forward” (light lysates from unexposed cells, heavy lysates from UV exposed cells) and “flip” (heavy lysates from unexposed cells, light lysates from UV exposed cells) of ZRF1^{FLAG} purifications. Almost all proteins show a ratio ≤2 (shown by dotted red lines) indicating no identification of differentially bound proteins. (d) Tethered ZRF1 binds XPC. Immunofluorescence images showing interaction of XPC-GFP in cells with array bound ZRF1-LacR, confirming functionality of both expressed proteins. Scale bar: 5 µm. (e) Western blot showing knockdown of XPC in shXPC cells compared to control (shNMC). (f) Western blot showing knockdown of ZRF1 in shZRF1 cells compared to control (shNMC).
